# Do anxiety, depression, and intolerance of uncertainty contribute to social problem solving in adult women with anorexia nervosa?

**DOI:** 10.1002/brb3.1588

**Published:** 2020-04-09

**Authors:** Lot Sternheim, Unna Danner, Annemarie van Elburg, Amy Harrison

**Affiliations:** ^1^ Department of Clinical Psychology Universiteit Utrecht Utrecht The Netherlands; ^2^ Altrecht Eating Disorders Rintveld Zeist The Netherlands; ^3^ Trimbos Institute Netherlands Utrecht The Netherlands; ^4^ Department of Psychology and Human Development Institute of Education University College London London UK

**Keywords:** anorexia nervosa, cognitive interpersonal maintenance model, experimental measures, self‐report measures, social problem solving

## Abstract

**Introduction:**

Inefficient problem solving in the social domain may be one of the difficulties underlying the interpersonal challenges thought to maintain anorexia nervosa (AN). However, past studies have neglected to control for depression, anxiety, and intolerance of uncertainty (IU), which are known to contribute to social problem solving.

**Methods:**

This study aimed to investigate whether adults with AN would show differences in social problem solving on an experimental task (Means‐End Problem Solving; MEPS) and report differences in their attitudes (positive, negative) toward social problem solving and their use of social problem‐solving styles (rational, impulsive–careless, avoidant) on the Social Problem‐Solving Inventory Revised (SPSRI) compared to a non‐AN control group.

**Results:**

Seventy‐four adult women took part (30 with AN and 44 non‐AN controls), and data show that those with AN generated significantly less effective solutions on the MEPS (*d* = 1.96) reported overall poorer social problem solving on the SPSRI (*d* = 0.58), reporting more negative and less positive attitudes toward social problem solving, and less impulsive and more avoidant social problem‐solving styles. However, those with AN did not differ from controls in being able to rationalize social problems. Once depression (Beck Depression Inventory: BDI), state anxiety (State‐Trait Anxiety Inventory: STAI), and IU (Intolerance of Uncertainty Scale‐12; IUS‐12) were included as covariates, these differences were no longer significant, suggesting that comorbid depression, anxiety, and IU symptoms may contribute to social problem solving in AN.

**Conclusions:**

There was no specific effect of depression. Treating anxiety and IU might help to improve social problem solving and enable people with AN to be able to better access social support to aid their recovery.

## Graphical abstract

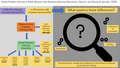

Compared to a non‐AN control group, women with AN showed poorer social problem solving abilities, as well as more dysfunctional social problem styles and attitudes. Anxiety and intolerance of uncertainty contributed to these social problem‐solving processes and outcomes.

## 1 INTRODUCTION

Problem solving is a higher order cognitive skill which supports the navigation of life's day‐to‐day challenges (Wallas, 1926). People with eating disorders (EDs) such as anorexia nervosa (AN), characterized by restriction of nutritional intake, significantly low body weight, an intense fear of weight gain and undue influence of body, weight, and shape on self‐evaluation (American Psychiatric Association, 2013) have been found to have difficulties with problem solving. They report higher avoidance and lower problem‐solving confidence than the general population (Soukup, Beiler, & Terrell, 1990). One domain of problem solving that might be particularly salient to people with AN, given the challenges they face in the interpersonal context (Treasure & Schmidt, 2013), is problem solving in the social context. Social problem solving is the process through which people attempt to identify or discover effective and adaptive solutions to problems they experience in their daily life (D'Zurilla, Nezu, & Maydeu‐Olivares, 2002). Effective social problem solving inherently requires good interpersonal skills. For individuals with AN, a lack of interpersonal skills is actually thought to play a central role in both the development and maintenance of AN (Arcelus, Haslam, Farrow, & Meyer, 2013; Carter, Kelly, & Norwood, 2012). In fact, interpersonal difficulties are thought to be so key to AN that these difficulties are targeted in recent treatment protocols (Maudsley Anorexia Nervosa Treatment for Adults; MANTRA, (Schmidt, Wade, & Treasure, 2014, Interpersonal Therapy for Eating Disorders; IPT‐ED; Rieger et al., 2010). These interpersonal difficulties may contribute to the high levels of social withdrawal and isolation that patients with AN often report. This social avoidance has been hypothesized to function as a way to reduce the negative emotions that are triggered by social interactions and interpersonal relationships (Treasure & Schmidt, 2013). Improving social problem solving may help those with AN navigate social interaction and interpersonal relationships and so reduce social withdrawal.

Despite the presence/relevance of interpersonal difficulties for individuals with AN, only a small number of studies have investigated social problem solving in AN. Three previous studies used the revised Social Problem Solving Inventory (SPSRI; D'Zurilla et al., 2002) self‐report measure to investigate social problem solving in people with AN. All three studies found, that compared to a non‐AN control group, people with AN report different attitudes toward problem solving than non‐AN controls, namely lower positive problem orientation and higher negative problem orientation. This means that people with AN report having a reduced constructive problem‐solving cognitive set, whereas unaffected peers take the approach that “whenever I have a problem, I believe it can be solved” (D'Zurilla et al., 2002). These studies also found that people with AN show differences in social problem‐solving styles, reporting a higher impulsive–careless style (i.e., a dysfunctional problem‐solving dimension characterized by active problem‐solving attempts that are impulsive, careless, hurried, and incomplete) and greater avoidance (an avoidance problem‐solving style refers to procrastination, passivity, or inaction, and a tendency to hold other people responsible for their problems and problem solving) than controls. However, those with AN show relative strengths in their ability to use a rational problem‐solving style and their scores did not differ from non‐AN controls on this subscale of the SPSRI (Paterson, Power, Yellowlees, Park, & Taylor, 2007; Patterson et al., 2010; Swanson et al., 2010). This suggests that those with AN report that they can logically think through and solve problems. However, one issue with these studies is that they rely on measuring social problem solving using a self‐report tool and do not measure what people with AN actually do when facing a real‐life problem. In other words, while these studies examine social problem‐solving attitudes, they did not assess social problem‐solving abilities, or whether these two constructs (i.e., attitudes versus abilities) relate to each other.

Two of these previous studies (Paterson et al., 2007; Patterson et al., 2010) also looked at associations between social problem solving attitudes and AN psychopathology. Findings confirm associations between AN symptoms and social problem solving, that is a more negative problem orientation and a more avoidant problem‐solving style was associated with more severe AN psychopathology (such as restrained eating and eating and weight concerns). However, rational problem solving and an impulsive–careless problem‐solving style were not significantly related to AN symptoms (Paterson et al., 2007; Patterson et al., 2010). Positive problem orientation was negatively associated with AN psychopathology in one study (Patterson et al., 2010), but not in the other (Paterson et al., 2007). This combination of low positive and high negative attitudes toward social problem solving alongside impulsive–careless and/or avoidant social problem‐solving styles has been described as maladaptive social problem‐solving by D'Zurilla and Nezu (1999), whose extensive work on social problem solving includes a wide range of participants, from university students to psychiatric inpatients.

Social problem solving ability has also been measured experimentally in one previous study in people with AN (Sternheim et al., 2012). This study used the Social Problem Resolution Task (Channon & Crawford, 1999) which assesses participants' ability to generate optimal solutions for social scenarios involving awkward everyday situations. Participants are asked to generate an effective solution for a hypothetical character in one of these awkward everyday situations, and secondly, they are asked what they themselves would do in this situation. While people with AN were able to generate optimal solutions for the hypothetical character that were as effective as those generated by a non‐AN control group, when asked what they themselves would do in that situation, they generated fewer optimal solutions. One further nonclinical study corroborates these findings and reported a negative association between problem‐solving ability measured using the Means‐End Problem Solving task (MEPS; Platt & Spivack, 1975) and eating pathology. In particular, a higher drive for thinness in female students was associated with the generation of less effective problem‐solving strategies (Ridout, Matharu, Sanders, & Wallis, 2015).

One issue with the evidence accrued so far is that the three previous self‐report studies (Paterson et al., 2007; Patterson et al., 2010; Swanson et al., 2010) are all from the same research group and the same clinical facility which could limit the strengths of any conclusions that might be drawn so far. A further problem with the current evidence on social problem solving in AN is that both the experimental study (Sternheim et al., 2012) and the three self‐report studies (Paterson et al., 2007; Patterson et al., 2010; Swanson et al., 2010) did not consider the role of, or, control for the impact of anxiety/depression on social problem solving, both pertinent comorbid features of AN (Godart, Flament, Perdereau, & Jeammet, 2002). This is an issue because previous work in clinical (i.e., chronic depressed patients) and nonclinical (student) samples has found that deficits in social problem solving are associated with anxiety disorders and depression (e.g., Klein et al., 2011). Regarding the relationship between depression and social problem solving, it has been hypothesized that social problem solving may be a consequence of state‐oriented rumination, a key feature of depression (Watkins & Baracaia, 2002). It has been suggested that this type of negative biased thinking then may be reflected in social problem‐solving processes, leading to ineffective social problem solving (in particular, avoidance styles and negative orientation) (Hasegawa, Kunisato, Morimoto, Nishimura, & Matsuda, 2018; Nolen‐Hoeksema, 1991).

High anxiety has also been linked to avoidant‐style cognitions and behaviors (Borkovec, Alcaine, & Behar, 2004), and also with negative thinking patterns (i.e., worry) (Haugh, 2006; Topper, Emmelkamp, & Ehring, 2010). Indeed, looking specifically at social problem‐solving measured by the SPSRI, positive and negative problem orientations were found to be significantly related to depression and anxiety; an avoidant social problem‐solving style was positively related to depression and anxiety, and the impulsive–careless social problem‐solving style was significantly related to depression (Haugh, 2006). Ma et al., (2017) found that participants with a body mass index in the obese range who had been diagnosed with depression reported less productive problem solving and a more negative attitude toward problem solving and endorsed a more avoidant style than those with lower levels of depression. Indeed, the large majority of studies examining social problem‐solving include measures of both depression and anxiety (Haugh, 2006).

Of note, conversely, positive affect facilitates creative problem solving (Isen, Daubman, & Nowicki, 1987). Looking at the broader anxiety literature, a theoretical model developed by Dugas, Gagnon, Ladoceur, and Freeston (1998) to explain anxiety incorporates concepts analogous to social problem‐solving, particularly problem orientation, and highlights the crucial role of intolerance of uncertainty (IU; Dugas et al., 1998; Ladouceur, Blaism, Freeston, & Dugas, 1998; Lecrubier et al., 1997). IU is a transdiagnostic personality trait and defined as the tendency to react negatively to uncertainty on cognitive, behavioral, and emotional levels (Dugas et al., 1998). Of course, social interactions inherently are uncertain, and responding to this uncertainty by, for example, avoidance behaviors or maladaptive cognitions is likely to interfere with effective social problem‐solving. Although IU is pertinent here because high IU is observed in AN (for an overview, see Kesby, Maguire, Brownlow, & Grisham, 2017; Sternheim, Konstantellou, Startup, & Schmidt, 2011), this potential confound has been neglected in previous investigations of social problem‐solving in AN. Therefore, this work is needed now because no previous studies in AN have controlled for depression, anxiety, and IU when measuring social problem‐solving. This will provide better knowledge of the specific contributions of these factors in social problem‐solving which could be used to optimize treatments. In other words, if indeed depression, anxiety, and IU contribute significantly to social problem solving, it may be worthwhile to incorporate treatment elements targeting these symptoms parallel to, or even before, social problem solving treatments.

Thus, the aim of this study was to explore whether people with AN show differences in their social problem‐solving skills relative to a non‐AN control group and whether these differences might be accounted for by depression, anxiety, and IU.

The first hypothesis was that people with AN would generate less effective social problem‐solving solutions than a non‐AN control group, measured using the MEPS, and report maladaptive attitudes and styles measured by the SPSRI.

The second hypothesis was that after controlling for depression, measured by the Beck Depression Inventory, anxiety, measured by the State‐Trait Anxiety Inventory, and IU, measured by the Intolerance of Uncertainty Scale (IUS‐12), and any differences between the AN and non‐AN control groups in social problem‐solving, measured using the MEPS and the SPSRI would no longer be significant. In other words, we expected that depression, anxiety, and IU would contribute to social problem‐solving in AN.

## 2 METHODS

### 2.1 Design

This quasi‐experimental study employed a cross‐sectional design.

### 2.2 Participants

Participants of any gender aged 18 or over were eligible for inclusion if they were able to provide informed consent and respond to the experimental materials in Dutch. Participants of any gender aged 18 or over were eligible for inclusion if they were able to provide informed consent and respond to the experimental materials in Dutch. Participants with AN were recruited from a specialist ED service (both in‐ and outpatients) in the Netherlands using opportunity sampling. Participants were informed about the study through flyers displayed in the service. The diagnosis of AN was confirmed by a Consultant Psychiatrist and Clinical Psychologist with ample experience in the treatment and diagnostic assessment of people with EDs on admission using DSM 5 criteria (American Psychological Association, 2013). Moreover, diagnosed were supported by questions from the Eating Disorder Examination Interview (Fairburn, 2008), the golden standard clinical interview for EDs. Patients diagnosed with other specified feeding or eating disorder (according to the DSM 5) were excluded.

Non‐AN controls were recruited using convenience sampling through flyers handed out at the University of Utrecht in the Netherlands and via advertising on social media. All non‐AN control participants were interviewed using the Mini International Neuropsychiatric Interview (M.I.N.I; Lecrubier et al., 1997) and excluded if they reported any current or lifetime eating disorder or any other psychiatric disorder.

### 2.3 Measures

The 40‐item State‐Trait Anxiety Inventory (Spielberger, 1983) was used to measure state and trait anxiety on a 4‐point Likert scale with higher scores indicating greater anxiety. Scores between 20 and 37 indicate no or low anxiety; scores between 38 and 44 indicate moderate anxiety and scores between 45 and 80 indicate high anxiety. The measure has good internal consistency (*α* = 0.86; Spielberger, 1983), and Cronbach's alpha was 0.68 for the state subscale and 0.71 for the trait subscale in this study. The Beck Depression Inventory (BDI; Beck & Beck, 1972) is a 13‐item self‐report measure of depressive symptoms and responses are obtained on a 4‐point Likert scale ranging from 0 to 3, with higher scores indicating greater depression. Internal consistency is good (*α* = 0.86; Beck, Steer, & Carbin, 1988), and Cronbach's alpha was 0.96 in this study. The 12‐item self‐report Intolerance of Uncertainty Scale (IUS‐12; Carleton, Norton, & Asmundson, 2007) measures intolerance of uncertainty of a 5‐point Likert scale ranging from 1 to 5, with higher scores indicating greater intolerance of uncertainty. Internal consistency is high (*α* = 0.94), and Cronbach's alpha in this study was 0.93. The IUS‐12 yields two subscales (prospective anxiety and inhibitory anxiety) and a total score. The total score was the main outcome measure used in this study. The 36‐item self‐report Eating Disorder Examination Questionnaire (Fairburn & Beglin, 1994) was used to measure ED pathology over the last 28 days on a 6‐point Likert scale. Internal consistency is good, ranging from 0.7 to 0.9 (Berg, Peterson, Frazier, & Crow, 2012), and Cronbach's alpha was 0.86 in this study. The EDE‐Q yields four subscales (restraint, eating concern, weight concern, and shape concern) and a global score. The outcome variable used in this study was the global EDE‐Q score.

### 2.4 Social problem‐solving tasks

#### 2.4.1 Means to end problem solving task

In this task, participants are presented with the beginnings and endings of six problems occurring in a social context and are asked to describe in writing the problem‐solving strategies that connect the beginnings with the endings of the problem scenarios (Platt & Spivack, 1975). Participants' responses are coded on a scale reflecting the effectiveness of their proposed solutions ranging from 0 to 7, where higher scores indicate more effective solutions. Following scoring procedures proposed by Williams, Barnhofer, Crane, and Beck (2005), responses were deemed effective if they maximize positive short‐ and long‐term consequences and minimize negative (short‐ and long‐term) consequences, both personally and socially. The scenarios were coded by two independent raters who obtained a 97% agreement rate, where agreement was considered as their independent scores either being consistent, or a maximum of one point higher or lower than the other rater (when there was more than one point higher or lower, the average score was used). The ratings for all six scenarios are combined to produce a total score which is the outcome variable used in this study.

#### 2.4.2 the Social Problem‐Solving Inventory (REVISED) (SPSRI)

The SPSRI a 25‐item self‐report measure which assesses on a 5‐point Likert scale ranging from 0 to 4: (a) attitudes toward social problem solving across two subscales: positive problem orientation (PPO; questions relating to a general cognitive set indicative of the tendency to view problems in a positive light, to see them as challenges rather than threats, and to be optimistic regarding the existence of a solution and one's ability to detect and implement effective solutions) and negative problem orientation (NPO; questions relating to a cognitive–emotional set that prevents effective problem solving); and (b) social problem‐solving styles: rational problem solving (RPS; questions relating to the tendency to use effective social problem‐solving techniques systematically and deliberately, including defining the problem, generating alternatives, evaluating alternatives, and implementing solutions and evaluating outcomes, impulsive–careless style (ICS; questions relating to the tendency to implement skills in an impulsive, incomplete, and haphazard manner) and avoidant style (AS; questions relating to dysfunctional patterns of social problem solving characterized by putting the problem off and waiting for problems to solve themselves) (D’Zurilla et al., 2002). Higher scores reflect greater intensity on a particular dimension. For this study, the scores for 15 negatively worded items on the negative problem orientation, impulsive style and avoidant style subscales were reversed to allow higher total scores to represent higher levels of social problem solving and this also permits the calculation of an overall Social Problem Solving score, thus higher scores across all scales indicate better problem solving. The SPSI‐R:SF possesses good levels of internal consistency and test–retest reliability, with internal consistency coefficients ranging from 0.73 for ICS; 0.80 for RPS; 0.82 for PPO; to 0.86 for both NPO and AS, shown in a previous validation study (Hawkins, Sofronoff, & Sheffield., 2009). This study also showed that test–retest coefficients were high with 0.79 for ICS; 0.81 for AS; 0.85 for both NPO and RPS; and 0.87 for PPO (*p* < .01).

### 2.5 Procedure

After hearing about the study and providing informed consent, participants were invited to meet the researcher to complete the measures. The participants with AN completed the measures in a quiet room at their specialist clinical facility and the non‐AN controls attended a research appointment at Utrecht University to complete the tasks. The test battery was the same for each participant, first they completed the questionnaires, subsequently they solved the MEPS. The study received ethical approval from the Institutional Review Board of the Committee for Scientific Research at Altrecht Mental Health Institute.

### 2.6 Data analysis

The data were analyzed using SPSS version 25. After checking that the data met assumptions of normality using histograms, box plots, and the Kolomogrov–Smirnov test, parametric tests were selected. Estimates of effect size are calculated using Cohen's *D* (Cohen, 1977), where 0.2 = small effect, 0.5 = medium effect, and 0.8 = large effect. *t* tests were used to examine whether there were group differences on the BDI, STAI, and IUS to understand whether to covary for these factors in the analysis.

Two models were built in order to test the hypotheses. First, multiple analysis of variance (MANOVA) was used to explore between‐group differences on the MEPS, the five subscales of the SPSRI and the overall SPSRI score. Group was entered as the independent variable, and the MEPS and SPSRI were entered as the dependent variables. Second, multiple analysis of covariance (MANCOVA) was used to explore whether any group differences remained after controlling for the proposed covariates of depression, anxiety, and IU. In this model, group was entered as the independent variable, the MEPS and SPSRI were entered as the dependent variables with state and trait anxiety (measured using the STAI) and IU (measured using the IUS) entered as covariates. Post hoc tests (ANOVAS) were performed and the p value was corrected using the Bonferroni correction (0.05/7, meaning that a *p* value ≤ .007 indicates the presence of a significant difference.

A power calculation conducted using GPower, based on data collected by Swanson et al., (2010), with power set at 80% to detect a medium‐sized difference (*d* = 0.5) or greater, and alpha set at 0.05, a minimum of 12 participants were required per group.

Discriminant function analysis was used to explore the relative contributions of all social problem‐solving variables to differentiating the AN and non‐AN control groups.

## 3 RESULTS

The final sample consisted of 74 women; 30 with AN and 44 non‐AN controls. Within the AN group, 16 participants had AN restrictive subtype and 14 had AN binge–purge subtype. The AN group had been unwell for a mean of 6.78 years (*SD* = 6.47), with a range of 1–27 years.

The groups were well matched in terms of age as shown in Table 1.

**Table 1:** Descriptive data for demographic and clinical variables for the eating disorder and noneating disorder control groups

Measure (mean, *SD*, 95% confidence interval)	All participant *n* = 74	Anorexia nervosa group *n* = 30	Nonanorexia nervosa controls *n* = 44	Test statistics
Age	24.72 (4.82)	24.03 (6.44)	25.18 (3.32).	*t*(72) = −1.15, *p *= .318, *d *= 0.24
	23.60–25.83	21.63–26.44	24.17–26.19	
BMI	19.94 (4.22).	16.01 (2.10)	22.62 (3.03).	*t*(72) = −10.36, *p *= ≤.001, *d* = 2.45
	18.96–20.92	15.23–16.80	21.70–23.54	
Years of illness	N/A	6.78 (6.47)	N/A	N/A
		4.37–9.20 Median = 4.50, IQR = 7 Minimum = 1 year, maximum = 27 years		
BDI total score	19.93 (6.91)	19.60 (7.03)	2.32 (2.51).	*t*(72) = 15.01, *p *= ≤.001, *d* = 3.55
	17.30–22.56	16.98–22.22	1.55–3.08	
EDE‐Q score	2.23 (1.25).	4.18 (1.26).	0.95 (0.93)	*t*(72) = 12.72, *p *≤ .001, *d* = 3.01
	3.75–4.23	3.71–4.65	0.66–1.23	
STAI state anxiety	51.36 (15.37).	65.30 (7.74)	41.86 (11.52).	*t*(72) = 9.73, *p *≤ .001, *d* = 2.39
	47.80–54.93	62.41–68.19	38.36–45.37	
STAI trait anxiety	52.69 (15.28)	66.23 (7.20)	43.45 (12.09).	*t*(72) = 9.25, *p *= ≤.001, *d* = 2.29
	49.15–56.23	39.78–47.13	39.78–47.13	
IUS‐12	32.96 (10.67).	41.43 (8.39)	27.18 (7.86)	*t*(72) = 7.46, *p *≤ .001, *d* = 1.76
	30.49–35.43	41.74–44.56)	24.79–29.57)	

Note

Median and IQR were included for length of illness because of the high standard deviation to provide an alternative measure of central tendency and dispersion.

Abbreviations: BDI, Beck Depression Inventory; BMI, body mass index; EDE‐Q, Global eating Disorder Examination Questionnaire; IQR, interquartile range; IUS‐12, Intolerance of Uncertainty Scale‐12; N/A, not applicable; STAI, State‐Trait Anxiety Inventory.

The MANOVA suggested a significant main of effect group for the social problem‐solving tasks (*V*(*s*)* *= 0.63, *F*(6, 65) = 18.227, *p* ≤ .001). As state and trait anxiety, depression, and IU differed significantly between the groups, as planned, they were included as covariates in the models described below.

Descriptive statistics for the ED and non‐AN control groups on the social problem‐solving styles and attitudes measure (SPSRI) and Means‐End Problem Solving Task (MEPS) outcome variables are provided in Table 2.

**Table 2:** Outcome data for the social problem‐solving styles and attitudes and means‐end problem solving task outcome variables for the anorexia nervosa and nonanorexia nervosa control groups

Measure (mean, SD, 95% confidence interval)	Anorexia nervosa group *n* = 30	Nonanorexia nervosa controls *n* = 44
Means‐end problem solving task	18.98 (7.55)	30.86 (4.83)
	16.11–21.85	29.37–32.35
Social problem solving styles		
SPSRI negative problem orientation	7.55 (4.79)	13.09 (3.16)
	5.73–9.37	12.12–14.07
SPSRI positive problem orientation	10.03 (4.63).	13.05 (2.94)
	8.28–11.79	12.14–13.95
Social problem solving attitudes		
SPSRI rational style	10.07 (3.53).	9.28 (3.63)
	8.72–11.41	8.16–10.40
SPSRI impulsive–careless style	14.79 (4.08)	11.84 (3.39)
	13.24–16.35	10.79–12.88
SPSRI avoidant problem solving style	12.03 (4.51)	15.00 (2.73)
	10.32–13.75	14.16–15.84
SPSRI total score	54.90 (12.61)	61.81 (10.35)
	50.19–59.61	58.65–64.94

SPSRI, Social Problem‐Solving Inventory Revised (D'Zurilla & Nezu, 1999 *;* 25‐item version).

The planned post hoc tests were conducted to explore the nature of these differences.

There was a significant main effect of group for the MEPS (*F*(1, 70) = 66.44, *p *≤ .001, *d* = 1.87). As shown in Table 2, the AN group showed significantly poorer social problem‐solving on this task than non‐AN controls. This difference was no longer significant when state and trait anxiety, depression, and IU were included as covariates (*F*(1, 66) = 3.47, *p *= .066), suggesting that the group differences are partly accounted for by comorbid psychopathology (depression, anxiety, and IU). IU (*F*(1, 66) = 6.87, *p *= .01), state anxiety (*F*(1, 67) = 0.42, *p *= .05), and trait anxiety (*F*(1, 67) = 5.50, *p *= .02) contributed individually to the model. While depression contributed to the model, its effect was not significantly independent (*F*(1, 66) = 1.02, *p *= .316).

There was a significant main effect of group for overall social problem‐solving attitudes and styles measured using the total of the SPSRI (*F*(1, 70) = 8.45, *p *= .005, *d *= 0.61). As shown in Table 2, those with AN reported greater maladaptive overall social problem‐solving attitudes and styles than non‐AN controls, reflecting poorer overall social problem solving. This difference was no longer significant when state and trait anxiety, depression, and IU were included as covariates (*F*(1, 66) = 1.56, *p *= .216), suggesting that the group differences are partly accounted for by comorbid psychopathology (depression, anxiety, and IU). IU contributed independently of the other covariates to the model: *F*(1, 67) = 8.67, *p *= .004. While state anxiety (*F*(1, 67) = 0.12, *p *= .914), trait anxiety (*F*(1, 67) = 0.04, *p *= .842), and depression (*F*(1, 66) = 0.69, *p *= .411) contributed to the model, their effects were not significantly independent.

There was a significant main effect of group for the negative problem orientation subscale of the SPSRI (*F*(1, 70) = 35.09, *p *≤ .001, *d* = 1.37). As shown in Table 2, the AN group reported a more negative attitude toward social problem solving than non‐AN controls. This difference was no longer significant when state/trait anxiety, depression, and IU were included as covariates (*F*(1, 66) = 3.47, *p *= .066), suggesting that the group differences are partly accounted for by comorbid psychopathology (depression, anxiety, and IU). IU (*F*(1, 66) = 50.68, *p *≤ .001) and trait anxiety (*F*(1,66) = 6.85, *p *= .011) contributed individually to the model. State anxiety (*F*(1, 66) = 0.4, *p *= .529) and depression: *F*(1, 66) = 0.19, *p *= .668 contributed to the model, but their effects were not significantly independent.

There was a significant main effect of group for the positive problem orientation subscale of the SPSRI (*F*(1, 70) = 11.42, *p *= .001, *d* = 1.79). As shown in Table 2, those with AN reported a less positive attitude to social problem solving than non‐AN controls This difference was no longer significant when state and trait anxiety, depression, and IU were included as covariates (*F*(1, 66) = 3.39, *p *= .07), suggesting that the group differences are partly accounted for by comorbid psychopathology (depression, anxiety, and IU). IU contributed independently of the other covariates to the model: *F*(1, 66) = 18.43, *p *≤ .001. While state anxiety (*F*(1, 66) = 0.06, *p *= .946), trait anxiety (*F*(1, 66) = 0.07, *p *= .786), and depression (*F*(1, 66) = 0.24, *p *= .623) contributed to the model, their effects were not significantly independent.

Both groups reported similar use of a rational problem‐solving style subscale of the SPSRI (*F*(1, 70) = 0.838, *p *= .363, *d *= 0.22). This nonsignificant effect remained when controlling for depression, anxiety, and IU (*F*(1, 66) = 0.07, *p *= .932). IU contributed independently of the other covariates to the model: *F*(1, 66) = 2.97, *p *= .090. While state anxiety (*F*(1, 66) = 0.86, *p *= .348), trait anxiety (*F*(1, 66) = 0.89, *p *= .348), and depression (*F*(1, 66) = 1.04, *p *= .312) contributed to the model, their effects were not significantly independent.

There was a significant main effect of group for the impulsive social problem‐solving style subscale of the SPSRI (*F*(1, 70) = 11.17, *p *= .001, *d *= 0.79). As shown in Table 2, the AN group reported significantly less use of impulsive problem solving than the non‐AN control group. This difference was no longer significant when state and trait anxiety, depression, and IU were included as covariates (*F*(1, 66) = 0.11, *p *= .738, suggesting that the group differences are partly accounted for by comorbid psychopathology (depression, anxiety, and IU). There was a statistical trend for IU (*F*(1, 66) = 3.48, *p *= .066), but none of the other variables contributed to the model with independent effects (state anxiety: *F*(1, 66) = 0.74, *p *= .93; trait anxiety: *F*(1, 66) = 0.10, *p *= .750; depression: *F*(1, 66) = 2.47, *p *= .122).

There was a significant main effect of group for the avoidant social problem solving subscale of the SPSRI (*F*(1, 70) = 12.10, *p *= .001, *d *= 0.81). As shown in Table 2, those with AN reported significantly greater use of avoidance problem‐solving styles than the non‐AN control group. This difference was no longer significant when state and trait anxiety, depression, and IU were included as covariates (*F*(1, 66) = 0.01, *p *= .932), suggesting that the group differences are partly accounted for by comorbid psychopathology (depression, anxiety, and IU). IU contributed independently of the other covariates to the model: *F*(1, 66) = 14.21, *p*≤.001. While state anxiety (*F*(1, 66) = 0.05, *p *= .809), trait anxiety (*F*(1, 66) = 0.19, *p *= .662), and depression (*F*(1, 66) = 1.56, *p *= .218) contributed to the model, their effects were not significantly independent.

In summary, the MANOVA, built to address the first hypothesis regarding between‐group differences on social problem solving, was significant: *F*(6, 65) = 18.23, *p *≤ .001, Wilks' Λ = 0.373; however, the second model built to test the impact of the depression, anxiety, and IU covariates on social problem solving was not significant: *F*(6, 61) = 1.42, *p *= .223, Wilks' Λ = 0.878).

The MANOVA was followed up with discriminant analysis, which revealed one discriminant function. This explained 100% of the variance, canonical *R^2^* = 1.68. The discriminant function analysis significantly differentiated the AN and non‐AN control groups Λ = 0.37, *X* ^2^(6) = 66.11, *p *≤ .001. The canonical variate correlation coefficients in Table 3 provide data on the relative contribution of each outcome variable to maximal separation of the groups and suggest that the MEPS and negative problem orientation were the variables contributing most to group separation, given that higher correlations contribute most to group separation (Bargman, 1970).

**Table 3:** Relative contributions of the social problem‐solving variables to group differentiation

Measure	Canonical variate correlation coefficient
Mean‐ends problem solving task	0.75
Negative problem orientation	0.55
Avoidant problem solving	0.32
Positive problem‐solving orientation	0.31
Impulsive problem‐solving	−0.31
Rational problem solving	−0.08

## 4 DISCUSSION

The key question this project sought to address was whether people with AN show differences in their social problem‐solving skills relative to a non‐AN control group and whether these differences might be accounted for by depression, anxiety, and IU.

The first hypothesis which was that people with AN would generate less effective social problem‐solving solutions than the non‐AN control group measured using the MEPS and show more maladaptive attitudes and styles on the SPSRI compared to controls was supported by the data. Those with AN provided significantly fewer effective solutions on the MEPS than controls. On the SPSRI, those with AN reported less positive and more negative attitudes to social problem solving than controls. Those with AN reported significantly lower use of impulsive–careless and significantly higher use of avoidant social problem‐solving styles. There were no differences for the rational social problem‐solving style.

The second hypothesis, which was that after controlling for depression, measured by the BDI, anxiety, measured by the STAI, and IU, measured by the IUS‐12, these differences between the AN and non‐AN control groups in social problem solving, measured using the MEPS and the SPSRI would no longer be significant, was also supported by the data. When depression, anxiety, and IU were included as covariates, the previously observed group differences were no longer significant. This suggests that the differences in social problem solving observed in and reported by the AN group relative to controls may partly underpinned by comorbid psychopathology such as depression and anxiety. Moreover, IU, a temperamental trait commonly observed in both individuals with anxiety and depression did seem to contribute to social problem solving. Further analyses suggest that in particular trait anxiety is implicated in negative orientation and avoidance solving style, while depression does not contribute independently to the social problem‐solving outcomes. Interestingly, IU seemed to contribute most strongly to all the social problem‐solving outcomes, highlighting the importance of IU to social problem solving in AN. In other words, alongside the importance of ED pathology, comorbid anxiety‐related psychopathology may contribute to issues commonly experienced by people with AN.

Of further interest, secondary analyses using discriminant function analysis pointed to particular deficits in generating less effective social problem‐solving solutions and negative problem orientation in AN. The post hoc discriminant function analysis added to the interpretation of the findings because it shows that differences in social problem solving help differentiate those with AN from non‐AN controls. This might be helpful in models assessing risk for the presence of AN symptoms. These results corroborate previous research (Paterson et al., 2007; Patterson et al., 2010; Sternheim et al., 2012; Swanson et al., 2010) demonstrating maladaptive social problem‐solving styles and attitudes in AN. Furthermore, these results add to the literature new knowledge that poor social problem solving in AN may be partly explained by comorbid psychopathology (depression and anxiety) and associated temperamental factors (IU).

The finding that people with AN strongly endorse a negative problem orientation and have a weaker positive problem orientation mirrors the low levels of self‐confidence and self‐esteem common in people with AN (Collin et al., 2016). It could be that some of the other factors contributing to more negative and less positive attitudes to social problem solving are self‐confidence and self‐esteem. Indeed, self‐esteem (Patterson et al., 2010), self‐competence (Paterson et al., 2007), and maternal bonding (Swanson et al., 2010) have been found to mediate relationships between social problem solving and ED pathology. In addition, the high levels of avoidant social problem‐solving style reported by the participants in this study are typical of the other avoidant styles of coping and avoidant behavior in AN, such as safety behaviors and cognitive avoidance strategies (Pallister & Waller, 2008; Sternheim et al., 2012).

The finding that participants with AN did not differ from controls on the rational problem‐solving style contribute to a broader literature suggesting a possible discrepancy between adequate cognitive abilities, and the actual inadequate implementation of generated strategies. For example, within the decision‐making literature, there are some studies showing inadequate decisions making in AN, while other studies fail to find any differences compared to HC groups (Danner et al., 2012; Guillaume et al., 2010). Similarly, Sternheim et al. (2012) found that while those with AN generated solutions comparable in effectiveness to HC participants when it regarded a hypothetical character, yet generated much poorer solutions when it regarded themselves. Indeed, this same phenomena has been observed in anxiety disorders where those with anxiety can generate effective solutions but implementation of these solutions fails (Dugas, Freeston, & Ladouceur, 1997).

The finding that those with AN had lower impulsive–careless social problem‐solving styles than controls was incongruent with previous research (Paterson et al., 2007; Patterson et al., 2010; Swanson et al., 2010). However, lower impulsivity is often observed in those with AN (Harrison, O'Brien, Lopez, & Treasure, 2010). It is possible that in our AN sample, there was less impulsivity characteristic than in the other 3 studies, however impulsivity as such was not assed in these studies or in the current study assessed impulsivity and it is thus difficult to draw any conclusions on this. There seem to be no obvious other differences between the current AN group and the groups in previous studies (i.e., on BMI or other parameters of severity).

A number of limitations should be considered when interpreting the findings of this study. An alternative interpretation of the data might be that a lack of power was the reason why the significant differences did not remain after controlling for the covariates. It would have been interesting to have explored whether there were differences between those with AN restricting and binge–purge subtypes on the social problem‐solving measures. However, the relatively small numbers within these subgroups made this problematic. Future work could involve recruiting larger numbers of participants with these variants of AN and other forms of ED, particularly as a study by Svaldi, Dorn, and Trentowska (2011) of women with binge eating disorder (BED) which administered the MEPS found those with BED generated significantly less effective solutions than non‐BED controls and that social problem solving was mediated by the severity of depression, body‐related self‐esteem, and perfectionism. The AN group was relatively heterogeneous in terms of length of illness (range of 1–27 years of illness) and may thus represent individuals with a more recent illness onset and those with a more severe and enduring illness. However, this may not be a fundamental issue when interpreting the findings because the regression models showed that ED symptoms themselves did not uniquely predict social problem solving. The study was open to all genders but only women took up the invitation to participate and future studies should explore how to recruit more males into the sample to understand whether the effects found can be generalized to other genders. The independent raters were not blinded for the participants groups when coding the scenarios, and interpretation biases can't be ruled out. It is furthermore possible that completing the self‐report measures before the experimental tasks might have increased anxiety in participants and in previous studies, we will consider counterbalancing the presentation of self‐report and experimental tasks measuring anxiety‐related constructs. It would have been helpful to have included an anxiety and/or depression control group to understand more about the transdiagnostic nature of their effects on social problem solving.

Results do suggest a specific role for IU, which corroborates IU as a transdiagnostic factor, relevant to AN (Kesby et al., 2017). More hypothetically, IU may be a shared operative mechanism that impacts social problem‐solving across various psychopathologies. A limitation of this study is that the data do not allow us to unravel the specific or overlapping effects of AN, IU, anxiety, and depression on social problem solving. It is also possible that these relationships are reciprocal and the underweight status of those with AN contributes to these pathologies and to the social problem solving difficulties (Jagielska & Kacperska, 2017), or that social problem difficulties maintain AN. An important avenue for further research, future studies should be of longitudinal nature and include larger sample sizes.

This study explored social problem solving in adults with AN, and it would be helpful to know whether the observed differences generalize to younger age groups, particularly as a qualitative study found adolescents with AN have reported difficulties around resolving interpersonal difficulties (Patel, Tchanturia, & Harrison, 2016). Future work would benefit from including a wider range of age groups and AN subgroups (including patients with varying levels of AN severity). Anxiety and depression were self‐reported, and it may be useful in future work to also include clinical diagnoses of these factors to further ascertain their role in social problem solving. Indeed, unfortunately, we did not obtain information about other diagnoses of the AN patients, and future studies should do so to obtain a more complete picture. Conversely, one strength of this study was that an experimental measure of social problem solving was included (the MEPS) which increased the ecological validity of the findings. Because this measure seemed sensitive to between‐group differences in people with and without AN, this may be a suitable tool to measure social problem solving in future studies within this population. Of note, although the MEPS is commonly used, the task is not validated in Dutch and conclusions should be interpreted with caution. Moreover, it could be that completing the SPSI‐R first may have influenced participant abilities on the experimental task.

These findings contribute to informing clinicians' formulations of the interpersonal maintenance factors which are important in leading evidence‐based treatments for AN (MANTRA, Schmidt et al., 2014 and Enhanced Cognitive Behavioral Therapy, Fairburn, 2008). These findings suggest that one way to help patients with AN to develop their social problem‐solving skills might be to treat their anxiety and IU. In fact, seeing that validated treatments for IU directly target anxiety (Boswell, Thompson‐Hollands, Farchione, & Barlow, 2013; McEvoy & Erceg‐Hurn, 2016), IU treatment may be an important additional component for those with AN, enhancing current AN treatments. Although depression did contribute to social problem‐solving outcomes, its contribution was not independent of anxiety and IU. This may be due to the phenomenological overlap between depression and anxiety (Goodwin, 2015), but could also be evidencing that while anxiety and anxiety processes (such as IU) are key to the development of AN, depression may be a consequence of starvation and as such have a different effect on social cognitive processes in AN.

Conversely, seeing the potentially reciprocal relationships between anxiety, depression, and AN, it may be possible that targeting social problem‐solving skills may also contribute to an amelioration in depression and anxiety. This new knowledge is important given how socially isolated those with AN are and the high degree of difficulty they report around their social functioning (Harrison, Mountford, & Tchanturia, 2014) and the importance of social support for recovery (McKnight & Boughton, 2009). It may also be that patients that come for treatment with high anxiety might be more likely to have social problem‐solving difficulties than patients with milder levels of anxiety and clinicians could adapt the support they offer when helping patients to rebuild their social support networks.

In conclusion, this study has shown that one of the factors underlying the interpersonal maintaining factors of AN (Fairburn, 2008; Treasure & Schmidt, 2013) might be suboptimal social problem solving skills and these inefficient skills may be due to anxiety and high IU, which could be ameliorated in treatment as a means of better supporting the social and cognitive skills of people with AN.

## CONFLICT OF INTEREST

None declared.

## AUTHOR CONTRIBUTIONS

LS involved in conceptualization, methodology, formal analysis, writing of the original draft, reviewing, and editing. UD involved in writing, reviewing, editing, and project administration.AE collected resources. AH involved in formal analysis, writing the original draft, reviewing, and editing.

## Data Availability

The data that support the findings of this study are available from the corresponding author upon reasonable request.
